# A data-constrained survival stratification model for surgically managed glioblastoma across public and real-world cohorts

**DOI:** 10.3389/fonc.2026.1908021

**Published:** 2026-10-07

**Authors:** Lin Zeng, DingJun Li, Yun Liao, Mengyu Du, Tao Wu, Yuxing Huang, Lisha Deng, Qizheng Li

**Affiliations:** 1 Department of Neurosurgery, Hospital of Chengdu University of Traditional Chinese Medicine, Chengdu, China; 2 Department of Neurology, Hospital of Chengdu University of Traditional Chinese Medicine, Chengdu, China

**Keywords:** CGGA, external validation, glioblastoma, nomogram, prognosis, real-world cohort, SEER, survival stratification

## Abstract

**Background:**

Glioblastoma (GBM) has highly heterogeneous postoperative survival. Although contemporary prognostic assessment ideally requires molecular, functional, and surgical-detail variables, these data are often unavailable in population-based and multicenter real-world datasets.

**Methods:**

Adults with histologically recorded GBM and tumor-directed surgery (resection or biopsy) in SEER formed the derivation cohort. A multivariable Cox model estimated 6-, 12-, and 18-month overall survival and was externally evaluated in CGGA and an independent institutional cohort without refitting. Because extent of resection, biopsy details, performance status, MGMT promoter methylation, IDH status, and treatment-regimen details were unavailable or non-harmonizable, the model was explicitly intended for registry-based survival stratification when contemporary core prognostic variables are absent.

**Results:**

The study included 3, 842 patients in the SEER derivation cohort, 512 in the CGGA validation cohort, and 96 in the institutional validation cohort. Age, marital status, year of diagnosis, tumor location, tumor size, radiotherapy, and chemotherapy were retained in the final model. The model showed acceptable discrimination and calibration in the derivation cohort and reproducible risk stratification in both validation cohorts. In the independent non-public institutional validation cohort, the 6-, 12-, and 18-month AUCs were 0.748, 0.721, and 0.694, respectively, with a Harrell’s C-index of 0.708, supporting reproducible risk stratification in a real-world clinical patient cohort.

**Conclusions:**

This multicohort study developed a registry-compatible survival stratification model for surgically managed glioblastoma for GBM using routinely available variables. The model may support survival estimation under data-constrained conditions, but should not be interpreted as a comprehensive neuro-oncology prognostic system or treatment decision-support tool.

## Introduction

Glioblastoma (GBM) is the most aggressive and lethal primary malignant brain tumor in adults (1, 2). The current standard treatment paradigm for eligible patients generally consists of maximal safe resection followed by radiotherapy with concomitant and adjuvant temozolomide (3). However, postoperative outcomes remain highly heterogeneous, and accurate survival estimation remains clinically important for patient counseling, follow-up planning, and risk stratification.

Contemporary prognostic assessment in GBM ideally requires integration of clinical, surgical, treatment-related, and molecular variables. Established and clinically relevant factors include age, performance status, extent of resection, tumor burden, radiotherapy, chemotherapy, MGMT promoter methylation, IDH status, and other molecular features (4, 5). However, many large population-based registries and multicenter real-world datasets do not consistently capture all of these variables (6, 7). Compared with many previously reported models, the present study was designed to emphasize cross-cohort harmonization of routinely available postoperative variables, the inclusion of tumor size and anatomical location, and external validation across both public and non-public real-world datasets. These studies have shown that integrating multiple variables may provide better discrimination than isolated predictors or conventional grouping strategies (8). At the same time, the contemporary pathological classification of diffuse gliomas increasingly emphasizes the importance of molecular markers in disease definition and prognostic assessment (9). In particular, extent of resection, KPS or other performance-status measures, MGMT promoter methylation, IDH status, biopsy-specific details, radiotherapy dose, and chemotherapy-regimen information are often unavailable or not harmonizable across datasets.

Several prognostic models and nomograms have been developed for glioma or glioblastoma, but their clinical interpretation is often affected by variable availability, cohort selection, and differences in validation strategy. Many models are developed within a single database, and fewer have been externally validated across both public and non-public real-world cohorts. A model based on harmonizable variables may not replace molecularly integrated prognostic systems, but it can help determine whether routinely available information retains reproducible prognostic value across heterogeneous datasets (10–12).

Accordingly, this multicohort retrospective study aimed to develop and externally validate a data-constrained survival stratification model for surgically managed glioblastoma for GBM using routinely available harmonizable variables. Postoperative GBM was defined as histologically confirmed GBM treated with tumor-directed surgery, including either resection or biopsy only. Because extent of resection, biopsy-specific details, KPS, MGMT promoter methylation, IDH status, and detailed treatment-regimen information were unavailable across harmonized cohorts, the model was designed as a pragmatic survival stratification framework rather than a comprehensive neuro-oncology prognostic system or treatment decision-support tool.

## Methods

### Study design and data sources

This multicohort retrospective study was conducted to develop and externally validate a data-constrained survival stratification model for surgically managed glioblastoma using routinely available and harmonizable variables. The primary objective was to evaluate whether a registry-compatible model could provide reproducible survival risk stratification across public and non-public external cohorts under conditions where key molecular, functional, and surgical-detail variables were unavailable. The derivation cohort was obtained from the Surveillance, Epidemiology, and End Results (SEER) database, a population-based cancer registry program sponsored by the National Cancer Institute. External validation was performed in two independent cohorts: a public external validation cohort from the Chinese Glioma Genome Atlas (CGGA) database and a non-public real-world institutional cohort from the Hospital of Chengdu University of Traditional Chinese Medicine. The SEER and CGGA datasets are publicly available and de-identified.

### Study population

Patients with histologically diagnosed glioblastoma who underwent tumor-directed surgical management were eligible for inclusion. In the context of this study, “postoperative GBM” refers to patients who had undergone either tumor resection or biopsy only before survival follow-up and postoperative treatment assessment. Detailed information regarding extent of resection or biopsy subtype was unavailable across the harmonized datasets and therefore could not be further analyzed. In the derivation cohort, adult patients were identified from the SEER database according to predefined eligibility criteria. Patients were included if they met the following criteria: (1) age ≥18 years; (2) histological diagnosis of glioblastoma; (3) receipt of tumor-directed surgery; (4) available overall survival data and follow-up information; and (5) available baseline clinicopathological and treatment-related variables. Patients were excluded if they had missing survival time, survival time equal to 0 months, incomplete information on postoperative radiotherapy or chemotherapy, or missing key baseline variables required for model construction. Similar eligibility criteria were applied to the CGGA external validation cohort, and only patients with available survival information, postoperative treatment data, and essential baseline variables were included in the final validation analysis.

Patients with survival time recorded as 0 months were excluded because such records could reflect same-month registration artifacts, incomplete follow-up resolution, or insufficient temporal information for time-to-event modeling. We acknowledge that this approach may also exclude a small number of perioperative or very early deaths, which should be considered when interpreting survival estimates.

Missing-data handling. Eligibility required complete information for survival, treatment, and the key baseline variables needed to apply the frozen model; consequently, primary analyses used complete cases for these variables. Tumor size was handled differently because an “unknown” category was explicitly recorded and sufficiently common; it was retained as a modeled registry state. Predictor-level pre-analysis missingness and analytic-cohort completeness are summarized in **Supplementary Table S1**. These choices may induce selection bias if missingness or unknown size is prognostic.

### Variable definition

Baseline variables included age at diagnosis, sex, marital status, year of diagnosis, tumor location, laterality, tumor size, radiotherapy, and chemotherapy. Age was analyzed as a continuous variable in regression modeling and was categorized for descriptive analyses when appropriate. Marital status was grouped as married or unmarried. Tumor location was categorized as frontal lobe, temporal lobe, parietal lobe, occipital lobe, or overlapping lesion/other. Tumor size was categorized as <40 mm, 40–59 mm, ≥60 mm, and unknown. Treatment-related variables included postoperative radiotherapy and chemotherapy, both coded as yes or no. In addition, adjuvant treatment patterns were classified into four groups: surgery alone, surgery plus radiotherapy, surgery plus chemotherapy, and surgery plus chemoradiotherapy. Because this was a multicohort study designed around cross-dataset harmonization, model construction was restricted to variables that were consistently available and comparably coded across the derivation and validation cohorts. Extent of resection, biopsy subtype, KPS or other performance-status measures, MGMT promoter methylation, IDH status, radiotherapy dose or fractionation, and chemotherapy-regimen details were not excluded because they were clinically unimportant; rather, they were unavailable, inconsistently recorded, or not harmonizable across all cohorts. Therefore, these variables could not be incorporated into the final registry-compatible model.

### Cross-cohort variable harmonization

To improve comparability across the SEER, CGGA, and institutional cohorts, variables were harmonized according to common definitions that could be consistently derived from all three datasets. Age was retained as a continuous variable in regression modeling and categorized descriptively when appropriate. Marital status was harmonized as married versus unmarried. Tumor location was grouped into frontal, temporal, parietal, occipital, and overlapping lesion/other categories. Tumor size was harmonized into <40 mm, 40–59 mm, ≥60 mm, and unknown. Radiotherapy and chemotherapy were both harmonized as binary postoperative treatment variables (yes/no). Variables that could not be consistently defined across all cohorts, including extent of resection, performance status, MGMT status, IDH status, and detailed treatment-regimen information, were not included in the final harmonized model. For external validation, the diagnosis-year variable in the institutional cohort could not be perfectly aligned with the original SEER calendar-period categories because all institutional patients were treated in a later calendar period. Therefore, the diagnosis-year variable was mapped to the later-treatment-era category when applying the SEER-derived model to the institutional cohort. This procedure preserved the original model structure but should be interpreted as an approximate temporal-era harmonization rather than an exact replication of the SEER calendar-period coding.

GBM ascertainment reflected the diagnostic information available in each source and era rather than retrospective molecular reclassification. SEER cases were identified from registry histology records, CGGA cases from the diagnosis recorded in the source dataset, and institutional cases from the contemporaneous pathological diagnosis in the medical record. The study interval spans major changes in WHO classification, especially the 2016 and 2021 incorporation of IDH status into diffuse-glioma taxonomy. Because IDH status was unavailable or could not be harmonized, earlier cases may include tumors that would now be classified as astrocytoma, IDH-mutant, grade 4, whereas later institutional cases more closely reflect contemporary criteria. The three cohorts therefore cannot be assumed to be biologically equivalent, and external validation estimates cross-source transportability of the recorded clinical label rather than validation within a molecularly uniform disease entity.

### Outcome definition

The primary endpoint of the study was overall survival (OS), defined as the interval from diagnosis to death from any cause or last follow-up. Patients who were alive at the last follow-up were censored. In addition to overall survival, time-specific survival probabilities at 6, 12, and 18 months were used for prognostic model evaluation.

### Institutional external validation cohort

To further evaluate model generalizability in a real-world clinical setting, we additionally included an independent institutional retrospective cohort from the Hospital of Chengdu University of Traditional Chinese Medicine as a non-public external validation cohort. Consecutive adult patients with histologically confirmed glioblastoma who underwent tumor-directed surgical management between January 2019 and December 2025 were retrospectively screened from hospital records. Patients with missing survival outcome data or unavailable key variables required for model application were excluded. Clinical information, treatment records, and follow-up data were retrospectively extracted and reviewed for model validation. Baseline clinical variables were harmonized with those used in the derivation model as closely as possible, including age, sex, marital status, year of diagnosis, tumor location, tumor size, postoperative radiotherapy, and chemotherapy. Overall survival was defined in the same manner as in the SEER and CGGA cohorts.

### Statistical analysis

Continuous variables were expressed as mean ± standard deviation (SD) or median with interquartile range (IQR), as appropriate, and categorical variables were presented as counts and percentages. Baseline characteristics between the derivation and CGGA external validation cohorts were compared using the Student’s t-test or Mann–Whitney U test for continuous variables and the chi-square test for categorical variables, as appropriate.

Univariable Cox proportional hazards regression analyses were first performed to describe the association of each candidate variable with overall survival. Multivariable model construction was not based on univariable significance alone. Instead, variables were selected according to a combination of clinical relevance, prior literature, and cross-dataset availability/harmonizability, and variables considered clinically important within the constraints of consistent multicohort coding were retained in the multivariable Cox proportional hazards regression model. Hazard ratios (HRs) and 95% confidence intervals (CIs) were calculated. The proportional hazards assumption was assessed using Schoenfeld residuals before interpretation of the final Cox regression model. Age was modeled as a continuous predictor to preserve information and maintain cross-cohort comparability; we acknowledge that alternative nonlinear specifications may be explored in future model refinement.

A nomogram for predicting 6-, 12-, and 18-month overall survival was constructed from the prespecified multivariable Cox model. The complete coefficients, baseline survival values, linear-predictor equation, and risk-group rule are reported in **Supplementary Table S2**. Risk groups were defined according to the 33.3rd and 66.7th percentiles of the prognostic score in the SEER derivation cohort. Patients with scores below the 33.3rd percentile were classified as low risk, those with scores between the 33.3rd and 66.7th percentiles as intermediate risk, and those with scores above the 66.7th percentile as high risk. These cutoff rules were fixed and applied without re-estimation to the CGGA and institutional external validation cohorts. Discrimination was assessed using Harrell’s C-index and time-dependent AUC. Internal validation used 1, 000 bootstrap resamples; apparent performance, mean optimism, and optimism-corrected performance were calculated. External calibration was quantified using calibration-in-the-large and calibration slope with 95% bootstrap confidence intervals in addition to time-specific calibration plots. A sensitivity analysis refitted the model after excluding diagnosis year and applied the reduced frozen model to both external cohorts without refitting. Confidence intervals for external-validation performance were obtained from 1, 000 bootstrap resamples.

For reproducibility, **Supplementary Table S2** reports the complete Cox coefficients, reference categories, baseline survival at 6, 12, and 18 months, linear-predictor equation, and tertile-based risk-group derivation. **Supplementary Table S3** reports bootstrap correction, external calibration, confidence intervals, and the diagnosis-year sensitivity analysis.

## Results

### Patient selection and baseline characteristics

The study flowchart is presented in **Figure 1**. A total of 5, 126 patients with glioblastoma were initially identified from the SEER database. After excluding patients aged <18 years, those without histological confirmation, those who did not undergo tumor-directed surgery, and cases with missing survival or treatment information, 3, 842 surgically managed patients were included in the derivation cohort. In addition, 586 patients were initially screened for the CGGA external validation cohort, of whom 512 were ultimately eligible after excluding cases with missing survival data, incomplete postoperative treatment information, or unavailable baseline variables.

**Figure 1:**
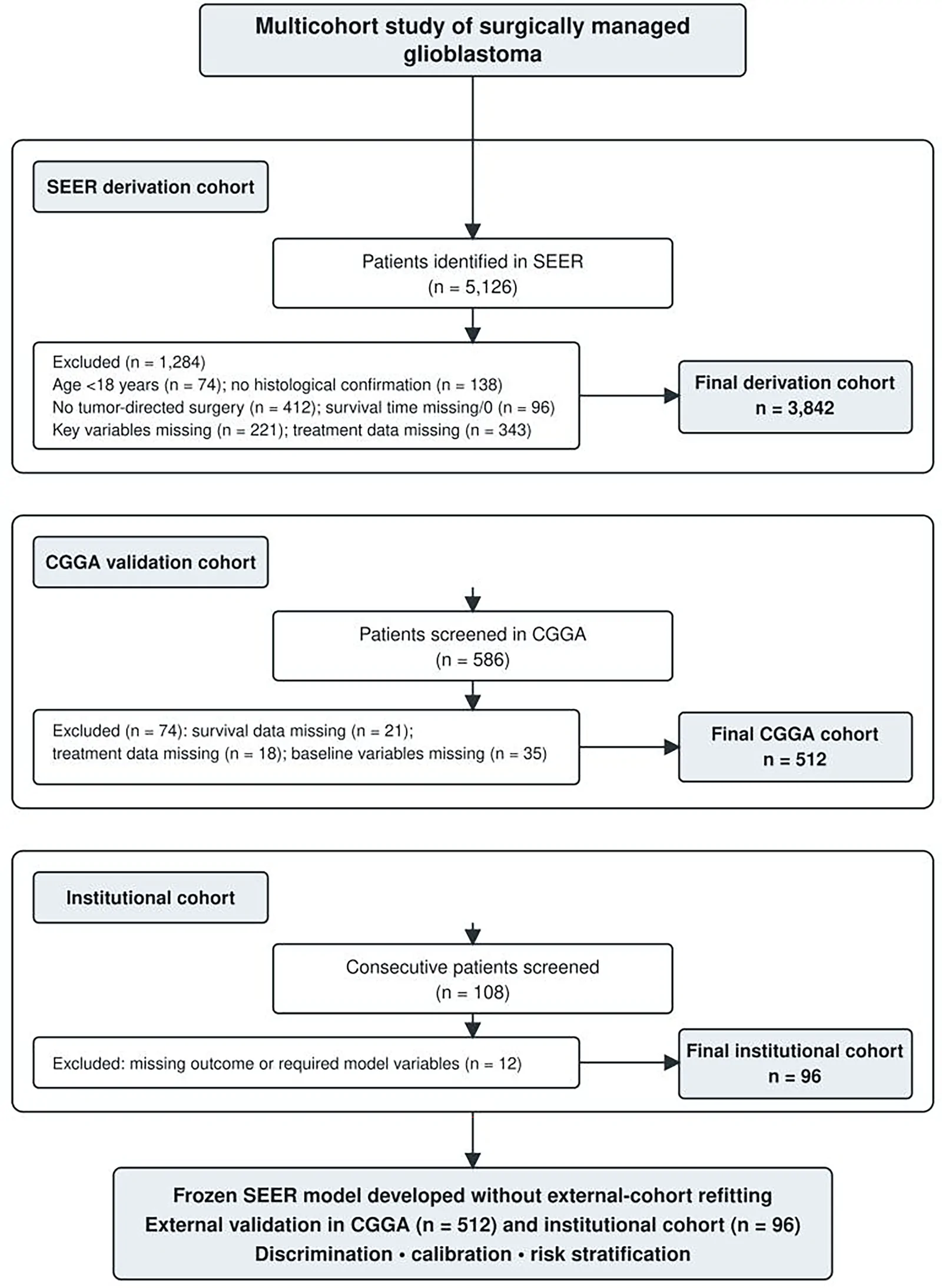
Flowchart of patient selection and study design.

For the institutional real-world external validation cohort, 108 consecutive patients with surgically managed glioblastoma were retrospectively screened, and 96 patients were finally included after excluding cases with missing survival outcome data or unavailable key variables required for model application.

After harmonization of comparable clinicopathological variables, baseline characteristics of the SEER derivation cohort and the CGGA external validation cohort are summarized in **Table 1**. Differences between the two cohorts were observed in several variables, including marital status, year of diagnosis, radiotherapy, chemotherapy, adjuvant treatment pattern, and median overall survival. Compared with the SEER cohort, the validation cohort included a higher proportion of patients diagnosed in more recent years and a greater proportion receiving radiotherapy, chemotherapy, and combined chemoradiotherapy. The mean age was 58.7 ± 13.4 years in the SEER cohort and 56.9 ± 12.8 years in the CGGA external validation cohort. Male patients accounted for 59.4% of the derivation cohort and 57.0% of the validation cohort. Median overall survival was longer in the validation cohort than in the derivation cohort (15.6 vs. 13.2 months). These between-cohort differences, particularly in treatment patterns, calendar period, and case mix, may have influenced absolute survival estimates and model calibration; however, the persistence of acceptable discrimination and risk-stratification performance across datasets supports the reproducibility of the selected harmonized predictors despite underlying cohort heterogeneity.

**Table 1:** Baseline characteristics of postoperative glioblastoma patients in the derivation and CGGA external validation cohorts.

Variable	SEER cohort (n = 3, 842)	CGGA external validation cohort (n = 512)
Age, years		
Mean ± SD	58.7 ± 13.4	56.9 ± 12.8
<65 years, n (%)	2, 436 (63.4)	349 (68.2)
≥65 years, n (%)	1, 406 (36.6)	163 (31.8)
Sex, n (%)		
Male	2, 281 (59.4)	292 (57.0)
Female	1, 561 (40.6)	220 (43.0)
Marital status, n (%)		
Married	2, 243 (58.4)	273 (53.3)
Unmarried	1, 599 (41.6)	239 (46.7)
Year of diagnosis, n (%)		
2004–2011	1, 694 (44.1)	126 (24.6)
2012–2019	2, 148 (55.9)	386 (75.4)
Tumor location, n (%)		
Frontal lobe	1, 182 (30.8)	171 (33.4)
Temporal lobe	1, 041 (27.1)	132 (25.8)
Parietal lobe	722 (18.8)	88 (17.2)
Occipital lobe	201 (5.2)	22 (4.3)
Overlapping lesion/other	696 (18.1)	99 (19.3)
Laterality, n (%)		
Left	1, 731 (45.1)	225 (43.9)
Right	1, 862 (48.5)	256 (50.0)
Bilateral/midline/other	249 (6.5)	31 (6.1)
Tumor size, mm, n (%)		
<40	1, 009 (26.3)	149 (29.1)
40–59	1, 468 (38.2)	201 (39.3)
≥60	973 (25.3)	109 (21.3)
Unknown	392 (10.2)	53 (10.4)
Radiotherapy, n (%)		
No	821 (21.4)	85 (16.6)
Yes	3, 021 (78.6)	427 (83.4)
Chemotherapy, n (%)		
No	1, 264 (32.9)	118 (23.0)
Yes	2, 578 (67.1)	394 (77.0)
Adjuvant treatment pattern, n (%)		
Surgery alone	512 (13.3)	41 (8.0)
Surgery + radiotherapy	752 (19.6)	77 (15.0)
Surgery + chemotherapy	309 (8.0)	44 (8.6)
Surgery + chemoradiotherapy	2, 269 (59.1)	350 (68.4)
Overall survival, months		
Median (IQR)	13.2 (7.4–21.8)	15.6 (9.1–24.7)
Vital status, n (%)		
Alive	618 (16.1)	98 (19.1)
Dead	3, 224 (83.9)	414 (80.9)

Continuous variables are presented as mean ± standard deviation (SD) or median (interquartile range, IQR), and categorical variables are presented as number (percentage). Because the derivation and validation cohorts were obtained from different public databases and a real-world institutional cohort, variables were harmonized for comparative presentation across datasets.

### Univariable and multivariable Cox regression analyses

Univariable and multivariable Cox regression analyses for overall survival in the derivation cohort are shown in **Table 2**. In univariable analyses, older age, unmarried status, larger tumor size, earlier year of diagnosis, absence of radiotherapy, and absence of chemotherapy were all significantly associated with worse overall survival. Certain tumor locations, particularly parietal lobe and overlapping lesion/other locations, were also associated with an increased mortality risk in unadjusted analyses.

**Table 2:** Univariable and multivariable Cox regression analyses for overall survival in the SEER cohort.

Variable	Univariable HR (95% CI)	P value	Multivariable HR (95% CI)	P value
**Age**, **years**	1.018 (1.015–1.021)	<0.001	1.015 (1.012–1.019)	<0.001
Sex				
Female	Reference		Reference	
Male	1.041 (0.979–1.107)	0.197	1.032 (0.969–1.099)	0.322
Marital status				
Married	Reference		Reference	
Unmarried	1.184 (1.116–1.256)	<0.001	1.121 (1.054–1.194)	<0.001
Year of diagnosis				
2004–2011	Reference		Reference	
2012–2019	0.836 (0.789–0.886)	<0.001	0.901 (0.848–0.957)	<0.001
Tumor location				
Frontal lobe	Reference		Reference	
Temporal lobe	1.057 (0.980–1.141)	0.151	1.042 (0.966–1.124)	0.287
Parietal lobe	1.102 (1.013–1.198)	0.024	1.071 (0.984–1.165)	0.112
Occipital lobe	1.116 (0.955–1.304)	0.167	1.083 (0.925–1.268)	0.32
Overlapping lesion/other	1.146 (1.051–1.249)	0.002	1.098 (1.006–1.199)	0.036
Laterality				
Left	Reference		Reference	
Right	1.028 (0.968–1.093)	0.369	1.019 (0.959–1.084)	0.542
Bilateral/midline/other	1.129 (0.987–1.291)	0.077	1.084 (0.946–1.242)	0.246
Tumor size, mm				
<40	Reference		Reference	
40–59	1.143 (1.061–1.231)	<0.001	1.118 (1.037–1.205)	0.004
≥60	1.291 (1.191–1.399)	<0.001	1.236 (1.138–1.342)	<0.001
Unknown	1.082 (0.969–1.208)	0.159	1.047 (0.936–1.171)	0.417
Radiotherapy				
No	Reference		Reference	
Yes	0.612 (0.570–0.657)	<0.001	0.701 (0.651–0.755)	<0.001
Chemotherapy				
No	Reference		Reference	
Yes	0.534 (0.501–0.569)	<0.001	0.621 (0.580–0.665)	<0.001

HRs and 95% CIs were estimated using Cox proportional hazards regression models. Variables with clinical relevance or statistical significance in univariable analyses were included in the multivariable model.

Bold values indicate statistically significant results (P < 0.05).

In the multivariable model, age remained an independent adverse prognostic factor (HR = 1.015, 95% CI: 1.012–1.019, P < 0.001). Unmarried status was independently associated with poorer survival compared with married status (HR = 1.121, 95% CI: 1.054–1.194, P < 0.001). Patients diagnosed during 2012–2019 had better overall survival than those diagnosed during 2004–2011 (HR = 0.901, 95% CI: 0.848–0.957, P < 0.001). Compared with tumors smaller than 40 mm, tumors measuring 40–59 mm (HR = 1.118, 95% CI: 1.037–1.205, P = 0.004) and ≥60 mm (HR = 1.236, 95% CI: 1.138–1.342, P < 0.001) were independently associated with increased mortality. Overlapping lesion/other tumor locations were also associated with poorer prognosis relative to frontal lobe tumors (HR = 1.098, 95% CI: 1.006–1.199, P = 0.036).

Among treatment-related variables, radiotherapy (HR = 0.701, 95% CI: 0.651–0.755, P < 0.001) and chemotherapy (HR = 0.621, 95% CI: 0.580–0.665, P < 0.001) were both independently associated with improved survival. Sex and laterality were not independently associated with overall survival after adjustment. Assessment based on Schoenfeld residuals did not indicate major violation of the proportional hazards assumption for the final multivariable Cox model.

### Forest plot of independent prognostic factors

A forest plot illustrating the independent prognostic factors identified in the multivariable Cox regression model is shown in **Figure 2**. Older age, unmarried status, larger tumor size, and overlapping/other tumor location were associated with increased mortality risk, whereas more recent year of diagnosis, radiotherapy, and chemotherapy were associated with reduced mortality risk. Among all variables included in the multivariable model, chemotherapy and radiotherapy showed the strongest protective associations with postoperative survival.

**Figure 2:**
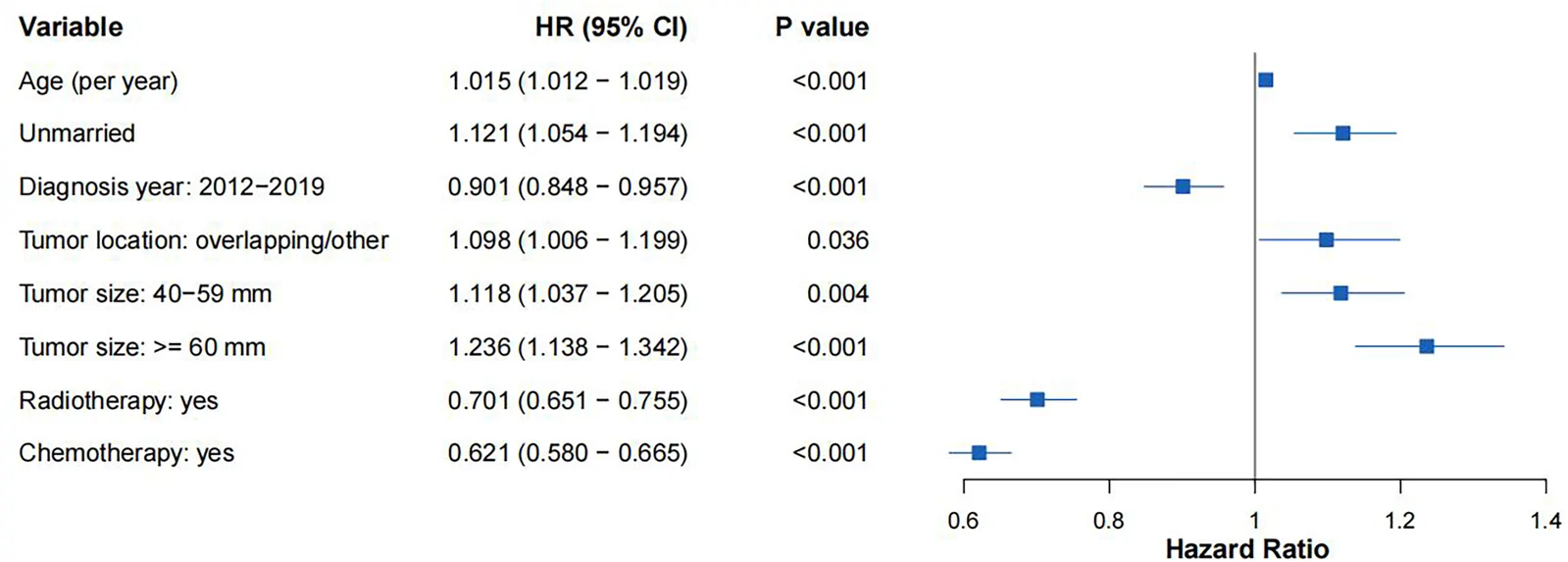
Forest plot of independent prognostic factors for overall survival in the SEER derivation cohort.

### Construction of the postoperative prognostic nomogram

Based on the independent prognostic factors identified in the multivariable model, a nomogram was constructed to predict 6-, 12-, and 18-month overall survival in patients with surgically managed glioblastoma (**Figure 3**). The nomogram incorporated age, marital status, year of diagnosis, tumor location, tumor size, radiotherapy, and chemotherapy. In terms of point allocation, the absence of chemotherapy and the absence of radiotherapy contributed the largest number of points, followed by older age and larger tumor size, suggesting that these variables had the strongest influence on predicted postoperative survival.

**Figure 3:**
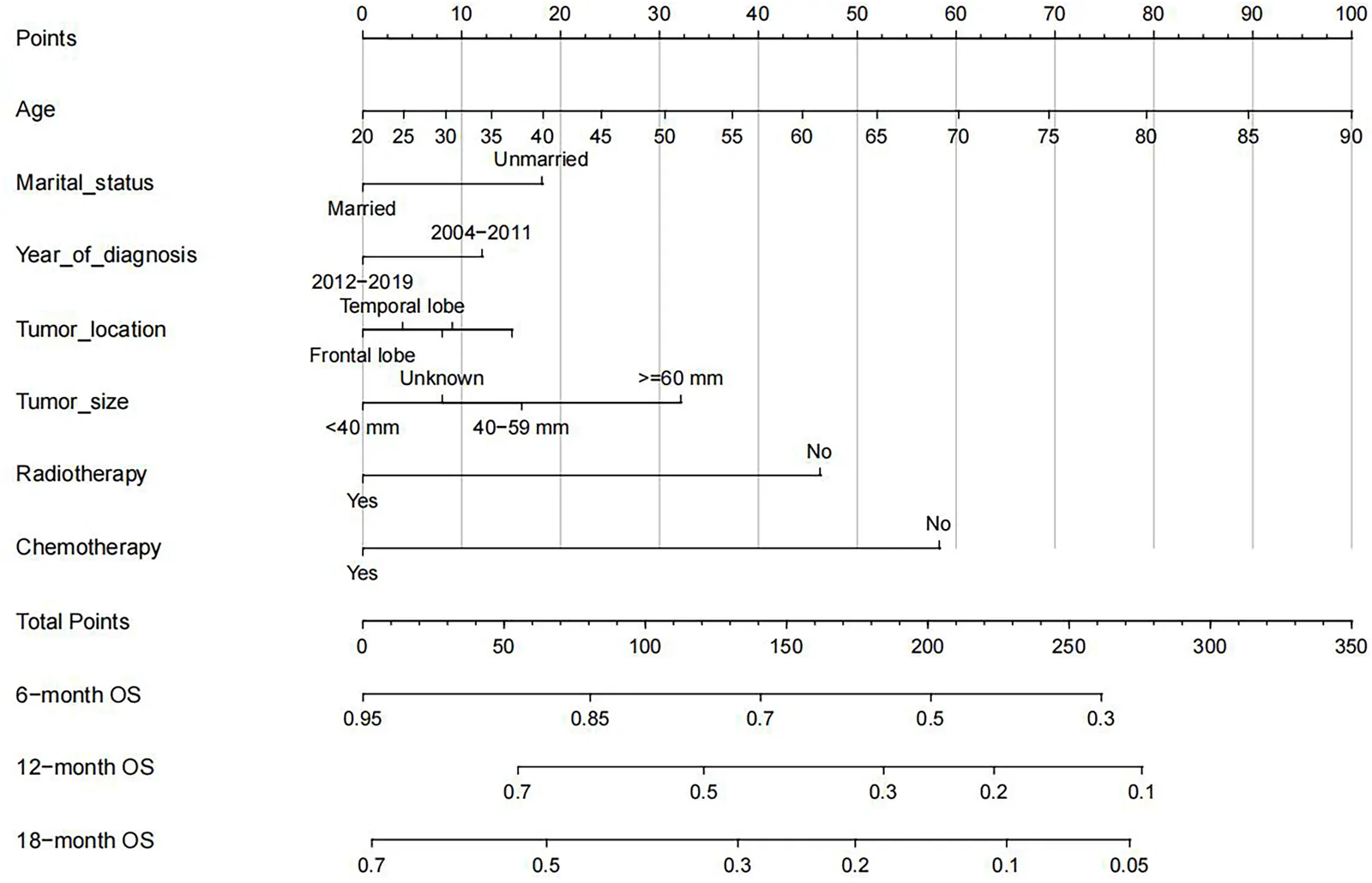
Nomogram for predicting 6-, 12-, and 18-month overall survival in patients with surgically managed glioblastoma.

### Discriminatory ability and calibration of the nomogram in the derivation cohort

The prognostic model demonstrated acceptable discriminatory ability in the derivation cohort. Time-dependent ROC analyses showed that the AUCs for predicting 6-, 12-, and 18-month overall survival were 0.781 (95% CI: 0.764–0.798), 0.756 (95% CI: 0.739–0.773), and 0.728 (95% CI: 0.709–0.747), respectively (**Figure 4A**).

**Figure 4:**
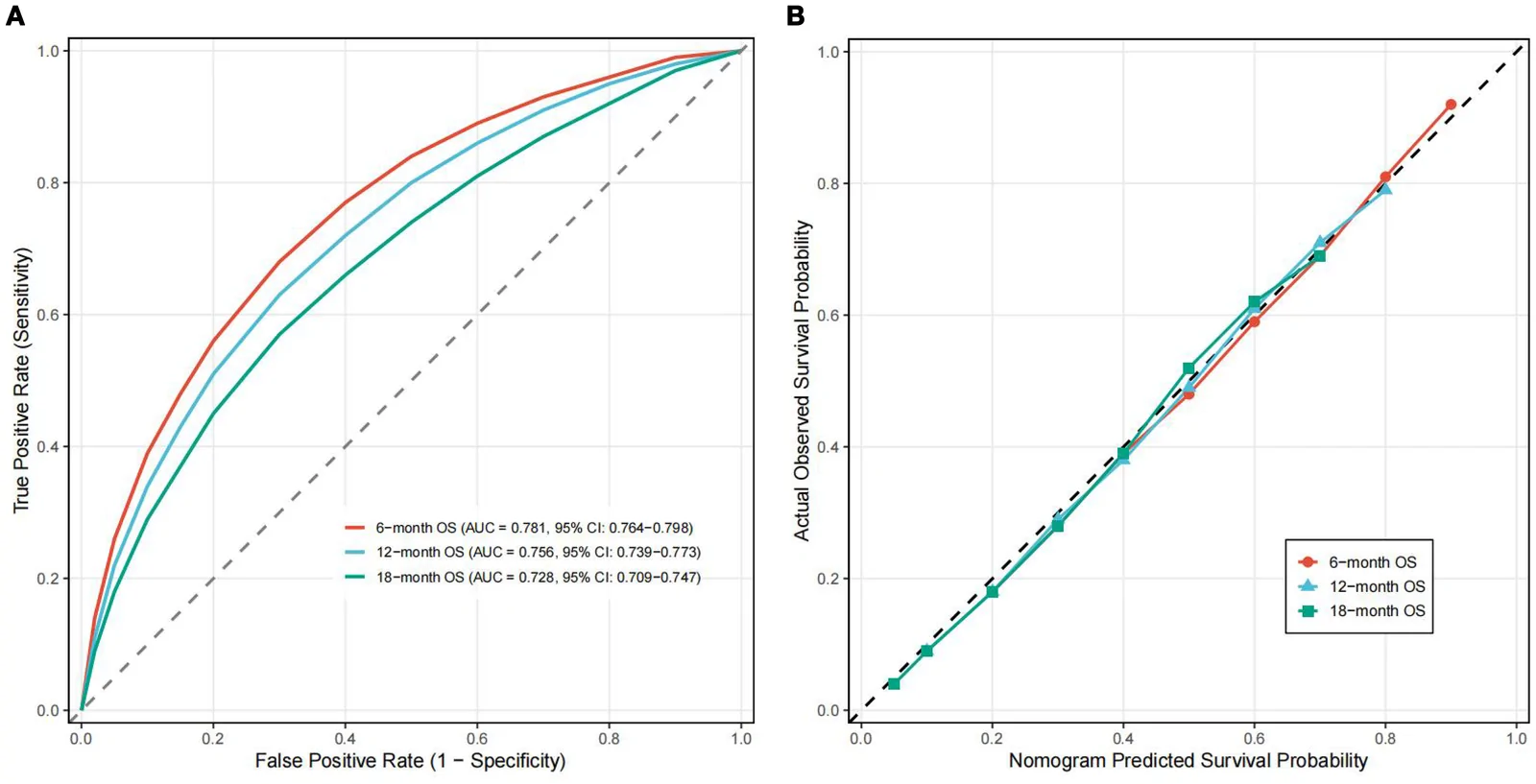
**(A)** Time-dependent ROC curves of the prognostic model for surgically managed glioblastoma for predicting 6-, 12-, and 18-month overall survival in the SEER derivation cohort. **(B)** Calibration curves of the prognostic model for surgically managed glioblastoma for predicting 6-, 12-, and 18-month overall survival in the SEER derivation cohort. The diagonal dashed line indicates ideal agreement between predicted and observed survival probabilities.

Calibration plots showed close visual agreement between predicted and observed survival at 6, 12, and 18 months (**Figure 4B**). Across 1, 000 bootstrap resamples, the apparent Harrell’s C-index was 0.729, mean optimism was 0.003, and the optimism-corrected C-index was 0.726. The corresponding optimism-corrected 6-, 12-, and 18-month AUCs were 0.779, 0.754, and 0.726, respectively, indicating limited apparent overfitting.

Decision-curve analysis is shown in **Figure 5** as an exploratory assessment of model-based risk thresholds. Because the model contains treatment variables and lacks several contemporary core prognostic factors, these curves do not establish treatment utility or justify changing care. They indicate only the mathematical net benefit of thresholding the predicted outcome under the assumptions of the analysis.

**Figure 5:**
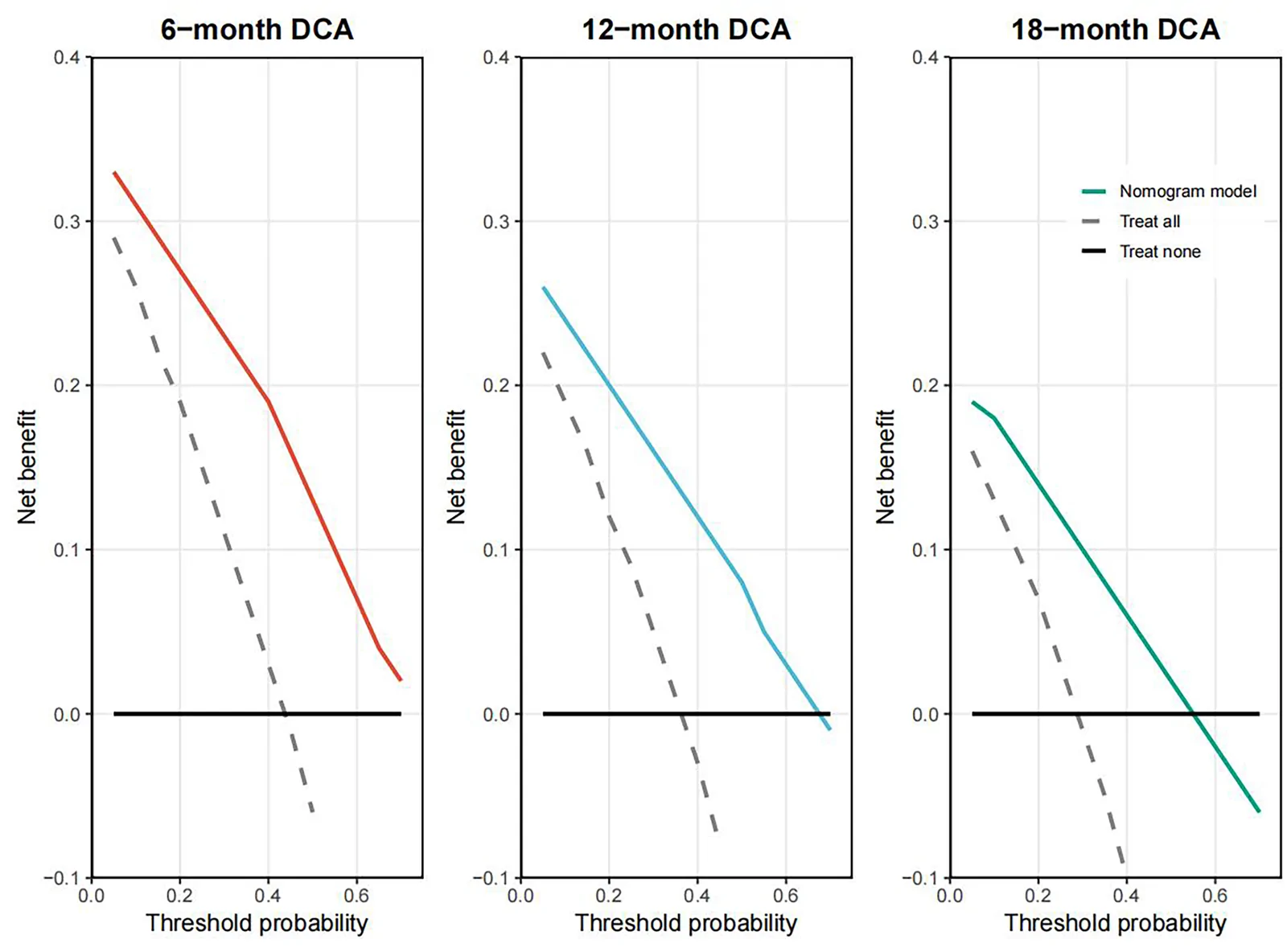
Exploratory decision-curve analysis of the prognostic model for predicting 6-, 12-, and 18-month overall survival in the SEER derivation cohort. The curves describe mathematical net benefit across threshold probabilities under the assumptions of the analysis and do not establish treatment utility or justify changes in care.

### Risk stratification and survival discrimination

Using the 33.3rd and 66.7th percentiles of the prognostic score in the SEER derivation cohort, patients were classified as low risk (n = 1, 286), intermediate risk (n = 1, 279), and high risk (n = 1, 277). The frozen tertile cutoffs were applied without re-estimation to CGGA and the institutional cohort. Kaplan–Meier curves separated across the three groups (log-rank P < 0.001; **Figure 6**). Median overall survival was 19.8 months (95% CI, 18.9–20.7), 12.7 months (95% CI, 12.1–13.3), and 7.4 months (95% CI, 7.0–7.8), respectively; mortality proportions were 71.6%, 84.1%, and 96.1%.

**Figure 6:**
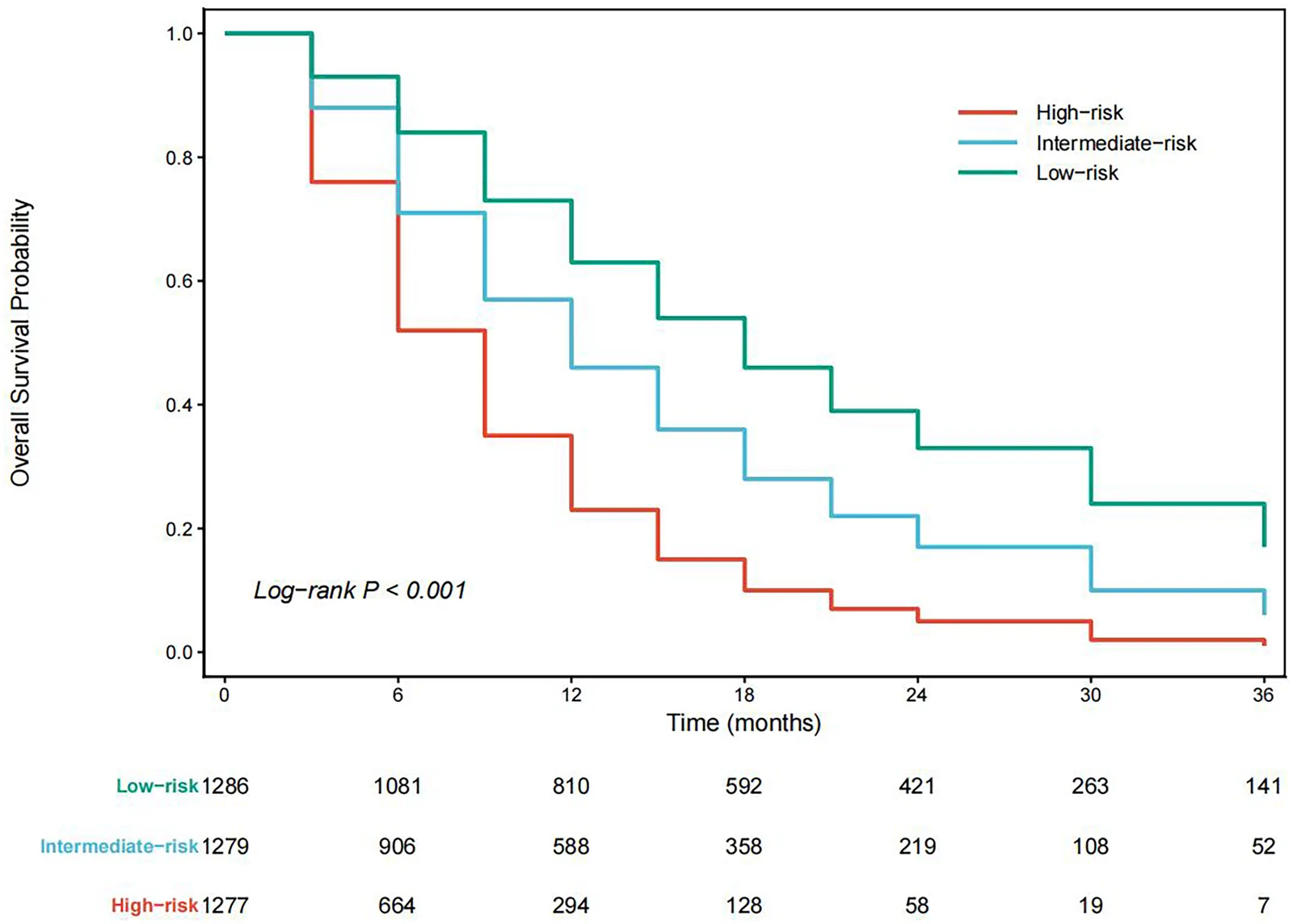
Kaplan–Meier survival curves according to model-defined risk groups in the SEER derivation cohort. Overall survival differed significantly across model-defined risk groups, with lower survival in the high-risk group and higher survival in the low-risk group. (log-rank P < 0.001).

### Sensitivity analysis excluding diagnosis year

After diagnosis year was removed, performance remained similar. In SEER, the reduced model yielded a C-index of 0.727 and 6-, 12-, and 18-month AUCs of 0.779, 0.753, and 0.725. Corresponding values were 0.706 and 0.752, 0.729, and 0.705 in CGGA, and 0.705 and 0.746, 0.719, and 0.692 in the institutional cohort. Absolute AUC changes were ≤0.003 relative to the primary model, suggesting that forced assignment of post-2019 institutional cases to the later SEER category had little influence on discrimination, although possible effects on absolute calibration remain.

The prognostic performance of the frozen nomogram was further evaluated in the CGGA external validation cohort (**Figure 7**). Kaplan–Meier curves differed among the predefined low-, intermediate-, and high-risk groups (log-rank P < 0.001). Median overall survival was 22.4 months (95% CI: 20.5–24.3), 14.6 months (95% CI: 13.2–16.0), and 8.3 months (95% CI: 7.5–9.1), respectively. This separation demonstrates prognostic discrimination but does not, by itself, demonstrate clinical usefulness.

**Figure 7:**
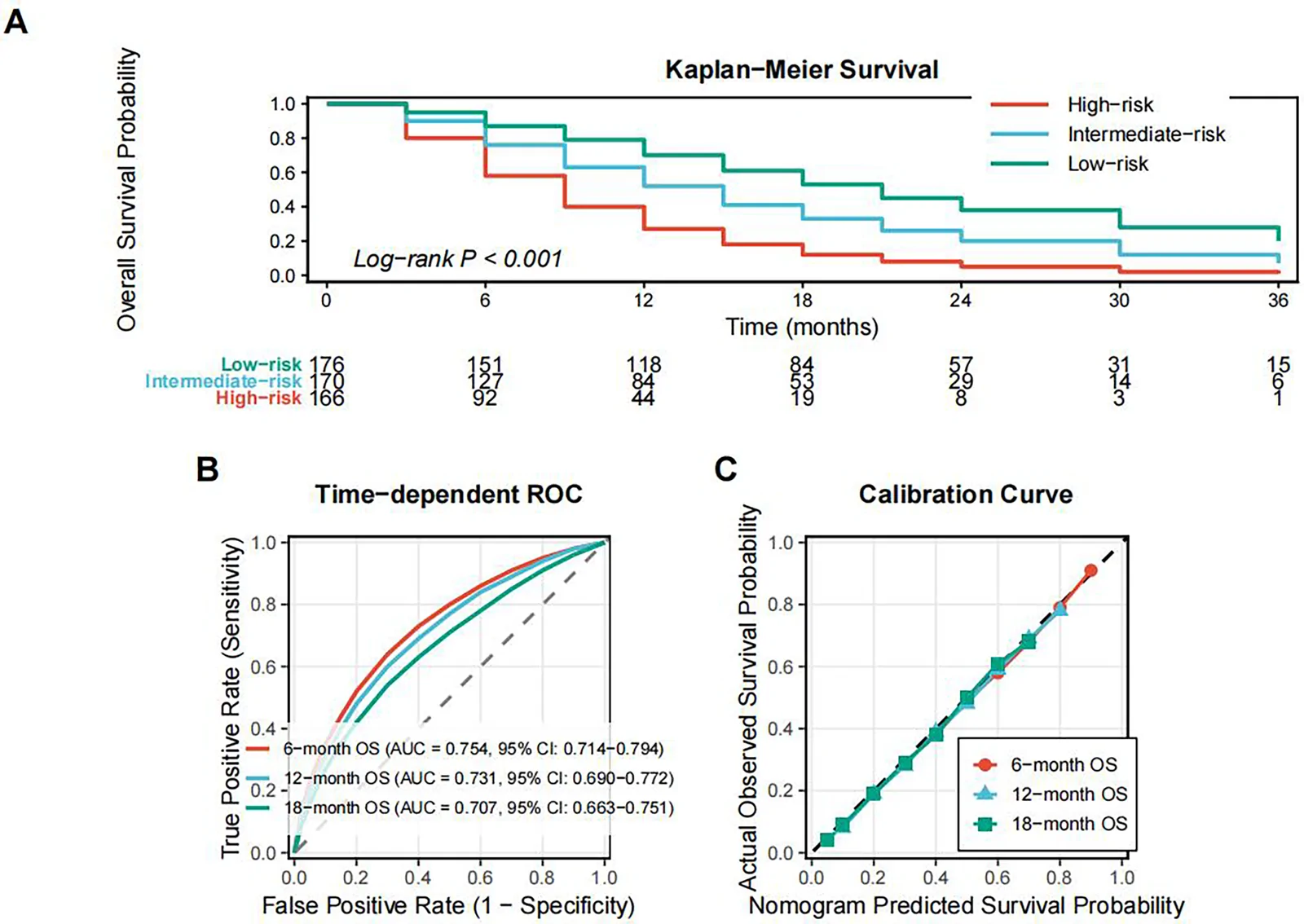
External validation of the prognostic model for surgically managed glioblastoma in the CGGA external validation cohort. **(A)** Kaplan–Meier survival curves according to model-defined risk groups. **(B)** Time-dependent ROC curves for predicting 6-, 12-, and 18-month overall survival. **(C)** Calibration curves showing agreement between predicted and observed survival probabilities.

The model retained moderate discrimination in the CGGA external validation cohort. The 6-, 12-, and 18-month AUCs were 0.754 (95% CI, 0.714–0.794), 0.731 (95% CI, 0.690–0.772), and 0.707 (95% CI, 0.663–0.751), respectively. Risk-group counts were 176, 170, and 166 in the low-, intermediate-, and high-risk groups. Calibration-in-the-large was 0.06 (95% CI, −0.04 to 0.16), and the calibration slope was 0.93 (95% CI, 0.82–1.05), consistent with generally preserved but not perfect calibration.

### Additional institutional validation

The institutional cohort included 96 patients with surgically managed glioblastoma; mean age was 57.6 ± 12.9 years, 55 (57.3%) were male, and median overall survival was 14.1 months (IQR, 8.2–22.9). When the frozen SEER-derived model was applied without refitting, time-dependent AUCs were 0.748 (95% CI, 0.666–0.830), 0.721 (95% CI, 0.624–0.818), and 0.694 (95% CI, 0.590–0.798) at 6, 12, and 18 months, respectively, and Harrell’s C-index was 0.708 (95% CI, 0.654–0.762). Kaplan–Meier curves separated across the predefined risk groups (low, n=32; intermediate, n=33; high, n=31). Calibration-in-the-large was 0.09 (95% CI, −0.12 to 0.30) and the calibration slope was 0.88 (95% CI, 0.62–1.15). Given only 96 patients, these estimates are imprecise and provide supportive—not definitive—evidence of transportability (**Figure 8**).

**Figure 8:**
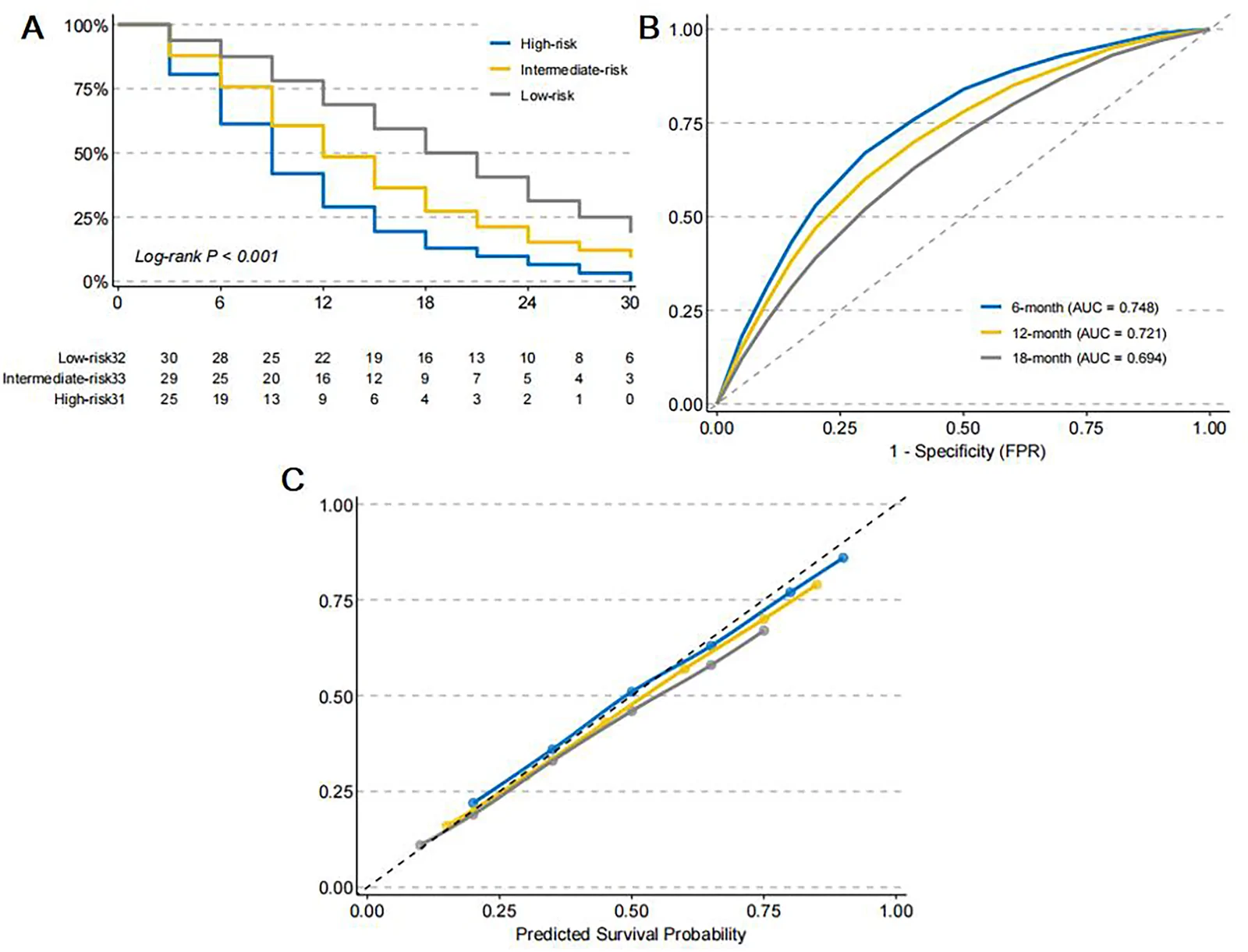
Real-world clinical external validation of the prognostic model for surgically managed glioblastoma in the institutional cohort. **(A)** Kaplan–Meier survival curves according to model-defined risk groups using the cutoff values derived from the SEER cohort. **(B)** Time-dependent ROC curves for predicting 6-, 12-, and 18-month overall survival. **(C)** Calibration curves showing the agreement between predicted and observed survival probabilities at 6, 12, and 18 months.

## Discussion

In this multicohort retrospective study, we developed and externally validated a data-constrained survival stratification model for surgically managed glioblastoma using routinely available and harmonizable variables. The model incorporated age, marital status, year of diagnosis, tumor location, tumor size, radiotherapy, and chemotherapy, and demonstrated acceptable discrimination, calibration, and reproducible risk stratification across the SEER derivation cohort, the public CGGA validation cohort, and a non-public institutional real-world cohort. Importantly, the present study should not be interpreted as establishing a comprehensive contemporary neuro-oncology prognostic system. Rather, it evaluates whether registry-compatible variables can provide reproducible postoperative survival stratification under conditions in which key molecular, functional, surgical-detail, and treatment-detail variables are unavailable or not harmonizable across datasets.

Glioblastoma remains the most aggressive primary malignant brain tumor in adults, and prognosis is poor despite multimodal treatment (13, 14). Since the landmark temozolomide era, maximal safe resection followed by radiotherapy with concomitant and adjuvant temozolomide has become the standard backbone of care (3, 15). In the pivotal and subsequent summary literature, the addition of temozolomide to postoperative radiotherapy improved median overall survival from approximately 12.1 months to 14.6 months, establishing combined chemoradiotherapy as the contemporary standard for eligible patients (3, 16). Population-based studies further suggested that survival modestly improved after the widespread adoption of temozolomide, although outcomes remain unsatisfactory overall (15, 17, 18). Against this background, transparent tools for descriptive survival stratification may be useful for registry research and patient communication, provided that discrimination is not conflated with treatment-effect prediction.

Our findings are broadly consistent with previous glioblastoma prognostic studies showing that age and treatment-related variables are among the most stable determinants of survival (6, 19). Older age has repeatedly been associated with poorer outcomes, likely reflecting a combination of adverse tumor biology, lower treatment tolerance, greater frailty, and a higher burden of comorbidity. Similarly, radiotherapy and chemotherapy have consistently emerged as favorable prognostic factors in registry-based and institutional series, which is in line with our current model (11). The association between more recent year of diagnosis and better survival is also biologically and clinically plausible (20). This temporal effect likely reflects improvements in neurosurgical techniques, perioperative care, access to standard chemoradiotherapy, and overall treatment standardization in the temozolomide era, rather than a calendar effect per se. These patterns are concordant with prior population-level analyses reporting survival gains for glioblastoma patients over time (21–23).

Despite these limitations, the model retained reproducible performance across independent validation settings. The CGGA cohort and the institutional cohort differed from the SEER derivation cohort in calendar period, treatment pattern, case mix, and data structure. Nevertheless, model-defined risk groups showed clear survival separation across cohorts, and the institutional validation cohort showed acceptable time-dependent AUCs and Harrell’s C-index. These findings suggest that routinely available variables still capture meaningful prognostic information. However, the institutional cohort was relatively small, and its validation results should therefore be interpreted as supportive rather than definitive evidence of generalizability.

The variables retained in the model are broadly consistent with known clinical patterns in glioblastoma. Older age was associated with worse survival, likely reflecting poorer treatment tolerance, greater frailty, comorbidity burden, and potentially adverse tumor biology. Larger tumor size and non-frontal or overlapping tumor locations may reflect greater tumor burden or more complex disease presentation. More recent year of diagnosis was associated with better survival, which may reflect improvements in perioperative care, radiotherapy planning, temozolomide-era treatment standardization, and supportive management over time. Radiotherapy and chemotherapy were associated with lower mortality in the model; however, these treatment variables should be interpreted as prognostic markers within an observational registry-based framework rather than as unbiased causal treatment effects.

Marital status should be interpreted as a context-dependent proxy rather than a biological prognostic factor. It may capture social support, caregiving availability, socioeconomic resources, treatment access, or unmeasured health status, and its meaning may differ across registry systems and cultures. A binary married/unmarried harmonization also obscures heterogeneous circumstances within the “unmarried” group. Its coefficient should therefore not be used for individual clinical judgment, and apparent cross-cohort reproducibility does not establish cross-cultural equivalence of the construct.

Radiotherapy and chemotherapy were retained as prognostic markers because they were consistently recorded across cohorts, but their coefficients must not be interpreted as causal treatment effects. Treatment receipt depends on performance status, extent of resection, comorbidity, disease burden, access to care, and survival long enough to initiate therapy—factors that were incompletely measured here. Modeling treatment as fixed from diagnosis also permits immortal-time bias. We therefore removed the within-risk-group treatment-pattern analysis from the principal manuscript because treatment variables also defined the risk groups, creating unavoidable circularity. A causal evaluation would require treatment start dates, time-dependent exposure modeling, and substantially richer confounder adjustment.

This study has several strengths. First, it used a relatively large population-based derivation cohort and two external validation cohorts, including a non-public real-world institutional cohort. Second, the model was deliberately restricted to variables that could be harmonized across datasets, improving transparency and external applicability in data-constrained settings. Third, model performance was evaluated using discrimination, calibration, and risk-stratification analyses rather than relying on a single performance metric. These features support the reproducibility of the model as a registry-compatible survival stratification tool.

Several limitations should be acknowledged. First, the retrospective complete-case design permits selection bias, residual confounding, and informative missingness. Second, absence of extent of resection, biopsy details, performance status, MGMT promoter methylation, IDH status, comorbidity, treatment timing, dose, and regimen substantially limits clinical interpretation. Recorded GBM diagnoses across 2004–2025 are not necessarily biologically equivalent under evolving WHO criteria; earlier cohorts may include IDH-mutant tumors that would now receive a different integrated diagnosis (24). Third, although diagnosis year could not be perfectly harmonized in the institutional cohort, the sensitivity analysis excluding diagnosis year yielded similar discrimination, suggesting that the forced temporal-category mapping had limited influence on model performance. Fourth, radiotherapy and chemotherapy coefficients are vulnerable to confounding by eligibility/indication and immortal-time bias and must not be read as treatment effects. Fifth, marital status is a culturally contingent proxy with limited individual-level meaning. Sixth, excluding survival recorded as 0 months may omit perioperative or very early deaths. Finally, the institutional cohort of 96 patients provides limited precision and only supportive evidence of transportability. Prospective validation with contemporary integrated diagnoses and richer clinical variables is required before clinical use.

## Conclusion

We developed and externally evaluated a data-constrained survival stratification model for surgically managed glioblastoma using routinely available variables. The frozen model showed moderate discrimination and separated model-defined risk groups across two external cohorts. These findings support registry-oriented prognostic stratification under missing-variable constraints, not equivalence to contemporary molecularly integrated assessment or clinical utility. The small institutional cohort, cross-era diagnostic heterogeneity, approximate diagnosis-year mapping, treatment-related bias, and absence of core prognostic variables preclude use for treatment selection or definitive individual prognosis.

## Data Availability

The original contributions presented in the study are included in the article/**Supplementary Material**. Further inquiries can be directed to the corresponding author.
