# Supplementary material for: A data-constrained survival stratification model for surgically managed glioblastoma across public and real-world cohorts
Source: Front Oncol. 2026 Oct 7;16:1908021. doi: 10.3389/fonc.2026.1908021 (PMC13683112; doi:10.3389/fonc.2026.1908021)
Supplement: Supplementary file 1 [file Table1.docx]

Supplementary Materials

Supplementary Table S1. Missing-data profile and analytic-cohort completeness

| Cohort | Pre-analysis missingness/exclusion information | Analytic N | Required predictors in analytic cohort |
| --- | --- | --- | --- |
| SEER | Surgery information 96; key baseline variables 221; radiotherapy/chemotherapy 343; tumor size unknown 392 (10.2%) | 3,842 | 0 missing by complete-case definition; tumor-size unknown retained |
| CGGA | Survival 21; postoperative treatment 18; essential baseline variables 35; tumor size unknown 53 (10.4%) | 512 | 0 missing by complete-case definition; tumor-size unknown retained |
| Institutional | Outcome or required model variables 12 | 96 | 0 missing by complete-case definition |

Supplementary Table S2. Frozen Cox model specification

| Predictor | HR | Coefficient ln(HR) |
| --- | --- | --- |
| Age, per year | 1.015 | 0.0149 |
| Unmarried | 1.121 | 0.1142 |
| Diagnosis 2012–2019 | 0.901 | -0.1043 |
| Temporal lobe | 1.042 | 0.0411 |
| Parietal lobe | 1.071 | 0.0686 |
| Occipital lobe | 1.083 | 0.0797 |
| Overlapping/other | 1.098 | 0.0935 |
| Tumor size 40–59 mm | 1.118 | 0.1115 |
| Tumor size ≥60 mm | 1.236 | 0.2119 |
| Tumor size unknown | 1.047 | 0.0459 |
| Radiotherapy | 0.701 | -0.3552 |
| Chemotherapy | 0.621 | -0.4764 |

Linear predictor = ΣβX using the coefficients above and the stated reference categories. Individual survival is S(t|X)=S0(t)^exp(LP). The baseline survival estimates were S0(6)=0.987, S0(12)=0.968, and S0(18)=0.946. Risk groups were defined by derivation-cohort score tertiles, producing 1,286/1,279/1,277 patients.

Supplementary Table S3A. Validation and sensitivity estimates

| Cohort/model | C-index | 6-month AUC (95% CI) | 12-month AUC (95% CI) | 18-month AUC (95% CI) |
| --- | --- | --- | --- | --- |
| SEER primary model | 0.729 | 0.781 (0.764–0.798) | 0.756 (0.739–0.773) | 0.728 (0.709–0.747) |
| SEER bootstrap-corrected | 0.726 | 0.779 | 0.754 | 0.726 |
| SEER model without diagnosis year | 0.727 | 0.779 | 0.753 | 0.725 |
| CGGA primary model | 0.709 | 0.754 (0.714–0.794) | 0.731 (0.690–0.772) | 0.707 (0.663–0.751) |
| CGGA model without diagnosis year | 0.706 | 0.752 | 0.729 | 0.705 |
| Institutional primary model | 0.708 (0.654–0.762) | 0.748 (0.666–0.830) | 0.721 (0.624–0.818) | 0.694 (0.590–0.798) |
| Institutional model without diagnosis year | 0.705 | 0.746 | 0.719 | 0.692 |

AUCs were estimated at 6, 12, and 18 months. Internal validation in the SEER derivation cohort was performed using 1,000 bootstrap resamples. The reduced sensitivity model excluded diagnosis year and was applied to both external cohorts without refitting. Calibration-in-the-large and calibration slopes were estimated in the external cohorts, with 95% confidence intervals obtained from 1,000 bootstrap resamples.

Supplementary Table S3B: External calibration results

| External cohort | Calibration-in-the-large (95% CI) | Calibration slope (95% CI) |
| --- | --- | --- |
| CGGA | 0.06 (−0.04 to 0.16) | 0.93 (0.82–1.05) |
| Institutional cohort | 0.09 (−0.12 to 0.30) | 0.88 (0.62–1.15) |
